# Diphenyl [(*S*)-1-phenylpropanamido]­phosphate

**DOI:** 10.1107/S1600536811034507

**Published:** 2011-08-27

**Authors:** Fahimeh Sabbaghi, Mehrdad Pourayoubi, Monireh Negari, Marek Nečas

**Affiliations:** a Department of Chemistry, Zanjan Branch, Islamic Azad University, PO Box 49195-467, Zanjan, Iran; b Department of Chemistry, Ferdowsi University of Mashhad, Mashhad, 91779, Iran; c Department of Chemistry, Faculty of Science, Masaryk University, Kotlarska 2, Brno CZ-61137, Czech Republic

## Abstract

The title compound, C_21_H_22_NO_3_P, was synthesized from the reaction of (C_6_H_5_O)_2_P(O)(Cl) and *S*-1-phenyl­propyl­amine (1:2 mole ratio) at 273 K, followed by removal of the *S*-1-phenyl­propyl­amine hydro­chloride by-product by dissolving in H_2_O. The P atom is located in a distorted tetra­hedral environment. The bond angles at the P atom vary from 99.51 (12) to 116.68 (12)°. The *sp* ^2^ character of the N atom is reflected by the C—N—P angle [120.9 (2)°]. The P=O group and the N—H unit adopt an *anti* orientation with respect to one another. In the crystal, adjacent mol­ecules are linked *via* N—H⋯O(P) hydrogen bonds into a one-dimensional arrangement running parallel to the *a* axis.

## Related literature

For background literature on phospho­ramidates having a C(=O)NHP(=O) skeleton, and the hydrogen-bond patterns and strengths, see: Toghraee *et al.* (2011 ▶); Pourayoubi *et al.* (2011 ▶). For a related phospho­ramidate with a P(=O)(O)_2_(N) skeleton, and its bond lengths and angles, see: Pourayoubi *et al.* (2010 ▶).
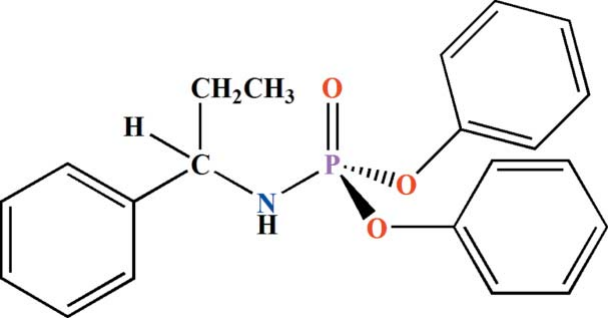


## Experimental

### 

#### Crystal data

C_21_H_22_NO_3_P
*M* *_r_* = 367.37
Orthorhombic, 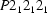

*a* = 5.4853 (3) Å
*b* = 8.1450 (11) Å
*c* = 41.162 (4) Å
*V* = 1839.0 (3) Å^3^
*Z* = 4
Mo *K*α radiation
μ = 0.17 mm^−1^
*T* = 120 K
0.40 × 0.20 × 0.20 mm

#### Data collection

Oxford Diffraction Xcalibur diffractometer with a Sapphire2 (large Be window) detector
Absorption correction: multi-scan (*CrysAlis RED*; Oxford Diffraction, 2009 ▶) *T*_min_ = 0.981, *T*_max_ = 1.000
4914 measured reflections
3000 independent reflections
2404 reflections with *I* > 2σ(*I*)
*R*_int_ = 0.029

#### Refinement

*R*[*F* ^2^ > 2σ(*F* ^2^)] = 0.052
*wR*(*F* ^2^) = 0.089
*S* = 1.07
3000 reflections
239 parameters
1 restraint
H atoms treated by a mixture of independent and constrained refinement
Δρ_max_ = 0.41 e Å^−3^
Δρ_min_ = −0.42 e Å^−3^
Absolute structure: Flack (1983 ▶), 1052 Friedel pairs
Flack parameter: −0.09 (14)

### 

Data collection: *CrysAlis CCD* (Oxford Diffraction, 2009 ▶); cell refinement: *CrysAlis RED* (Oxford Diffraction, 2009 ▶); data reduction: *CrysAlis RED*; program(s) used to solve structure: *SHELXS97* (Sheldrick, 2008 ▶); program(s) used to refine structure: *SHELXL97* (Sheldrick, 2008 ▶); molecular graphics: *Mercury* (Macrae *et al.*, 2008 ▶); software used to prepare material for publication: *enCIFer* (Allen *et al.*, 2004 ▶).

## Supplementary Material

Crystal structure: contains datablock(s) I, New_Global_Publ_Block. DOI: 10.1107/S1600536811034507/dn2713sup1.cif

Structure factors: contains datablock(s) I. DOI: 10.1107/S1600536811034507/dn2713Isup2.hkl

Additional supplementary materials:  crystallographic information; 3D view; checkCIF report

## Figures and Tables

**Table 1:** Hydrogen-bond geometry (Å, °)

*D*—H⋯*A*	*D*—H	H⋯*A*	*D*⋯*A*	*D*—H⋯*A*
N1—H1⋯O3^i^	0.84 (1)	2.25 (1)	3.077 (3)	167 (3)

Symmetry code: (i) 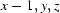.

**Fig. 1.**
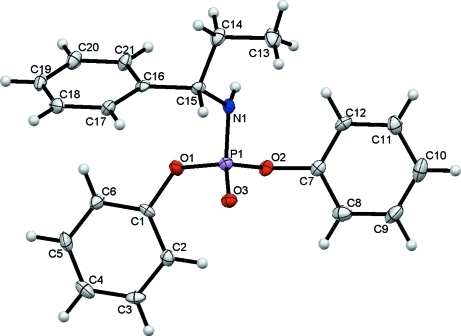
An ORTEP style plot and atom labeling scheme for the title compound. Displacement ellipsoids are given at 50% probability level and H atoms are drawn as small spheres of arbitrary radii.

**Fig. 2.**
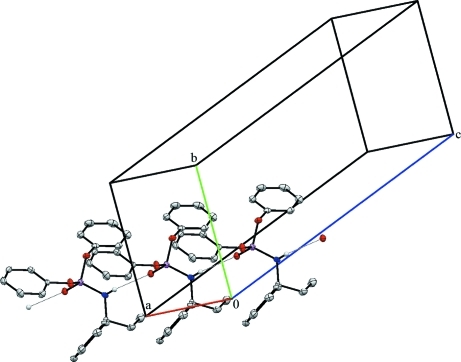
Part of the crystal packing of the title compound with the hydrogen bonds shown as dotted lines (the C—H hydrogen atoms are omitted for clarity).

## supplementary crystallographic information

### Comment

In recently published papers concerning phosphoramidate compounds having a C(═O)NHP(═O)(N)_2_ skeleton, the hydrogen bonds pattern (Toghraee *et al.*, 2011) and strengths (Pourayoubi *et al.*, 2011) were analyzed. In our continuing interest, we collected the structural data related to a new compound with a P(═O)(O)_2_(N) skeleton belonging to the phosphoramide family.

The molecular structure of the title compound is given in Fig. 1. The P═O, P—O and P—N bond lengths and the C—N—P and C—O—P angles are standard for this category of phosphoramidate compounds (Pourayoubi *et al.*, 2010).

In the crystal structure, molecules are linked *via* N—H···O(P) hydrogen bonds into extended chains running parallel to the *a* axis (Table 1, Fig. 2).

### Experimental

To a solution of (C_6_H_5_O)_2_P(O)Cl in chloroform, a solution of *S*-1-phenylpropylamine (1:2 mole ratio) in chloroform was added at 273 K. After 4 h of stirring, the solvent was removed and the obtained solid was washed with distilled water. Single crystals were obtained from a solution of the title compound in CHCl_3_/n-C_7_H_16_ after slow evaporation at room temperature.

### Refinement

All carbon bound H atoms were placed at calculated positions and treated as riding with their *U*_iso_ set to either 1.2*U*_eq_ or 1.5*U*_eq_ (methyl) of the respective carrier atoms; in addition, the methyl H atoms were allowed to rotate about the C—C bond. Nitrogen bound H atom was located in a difference Fourier map and its coordinates were refined using restraint on the N—H distance (0.85 (1) Å) with *U*_iso_ = 1.5*U*_eq_(N).

### Crystal data

**Table:**

C_21_H_22_NO_3_P	*F*(000) = 776
*M**_r_* = 367.37	*D*_x_ = 1.327 Mg m^−^^3^
Orthorhombic, *P*2_1_2_1_2_1_	Mo *K*α radiation, λ = 0.71073 Å
Hall symbol: P 2ac 2ab	Cell parameters from 1550 reflections
*a* = 5.4853 (3) Å	θ = 3.2–27.6°
*b* = 8.1450 (11) Å	µ = 0.17 mm^−^^1^
*c* = 41.162 (4) Å	*T* = 120 K
*V* = 1839.0 (3) Å^3^	Plate, colorless
*Z* = 4	0.40 × 0.20 × 0.20 mm

### Data collection

**Table:**

Oxford Diffraction Xcalibur diffractometer with a Sapphire2 (large Be window) detector	3000 independent reflections
Radiation source: Enhance (Mo) X-ray Source	2404 reflections with *I* > 2σ(*I*)
graphite	*R*_int_ = 0.029
Detector resolution: 8.4353 pixels mm^-1^	θ_max_ = 25.0°, θ_min_ = 3.2°
ω scan	*h* = −5→6
Absorption correction: multi-scan (*CrysAlis RED*; Oxford Diffraction, 2009)	*k* = −9→5
*T*_min_ = 0.981, *T*_max_ = 1.000	*l* = −48→48
4914 measured reflections	

### Refinement

**Table:**

Refinement on *F*^2^	Secondary atom site location: difference Fourier map
Least-squares matrix: full	Hydrogen site location: inferred from neighbouring sites
*R*[*F*^2^ > 2σ(*F*^2^)] = 0.052	H atoms treated by a mixture of independent and constrained refinement
*wR*(*F*^2^) = 0.089	*w* = 1/[σ^2^(*F*_o_^2^) + (0.0325*P*)^2^ + 0.0316*P*] where *P* = (*F*_o_^2^ + 2*F*_c_^2^)/3
*S* = 1.07	(Δ/σ)_max_ < 0.001
3000 reflections	Δρ_max_ = 0.41 e Å^−^^3^
239 parameters	Δρ_min_ = −0.42 e Å^−^^3^
1 restraint	Absolute structure: Flack (1983), 1052 Friedel pairs
Primary atom site location: structure-invariant direct methods	Flack parameter: −0.09 (14)

### Special details

**Table:**

Geometry. All esds (except the esd in the dihedral angle between two l.s. planes) are estimated using the full covariance matrix. The cell esds are taken into account individually in the estimation of esds in distances, angles and torsion angles; correlations between esds in cell parameters are only used when they are defined by crystal symmetry. An approximate (isotropic) treatment of cell esds is used for estimating esds involving l.s. planes.
Refinement. Refinement of *F*^2^ against ALL reflections. The weighted *R*-factor *wR* and goodness of fit *S* are based on *F*^2^, conventional *R*-factors *R* are based on *F*, with *F* set to zero for negative *F*^2^. The threshold expression of *F*^2^ > σ(*F*^2^) is used only for calculating *R*-factors(gt) *etc*. and is not relevant to the choice of reflections for refinement. *R*-factors based on *F*^2^ are statistically about twice as large as those based on *F*, and *R*- factors based on ALL data will be even larger.

### Fractional atomic coordinates and isotropic or equivalent isotropic displacement parameters (Å^2^)

**Table:**

	*x*	*y*	*z*	*U*_iso_*/*U*_eq_	
P1	1.02848 (15)	0.21930 (11)	0.13952 (2)	0.0144 (2)	
O1	1.0319 (4)	0.2594 (3)	0.10191 (4)	0.0190 (6)	
O2	0.9231 (4)	0.3876 (2)	0.15288 (5)	0.0159 (5)	
O3	1.2652 (4)	0.1671 (2)	0.15233 (5)	0.0168 (6)	
N1	0.8109 (5)	0.0886 (3)	0.14460 (7)	0.0122 (6)	
H1	0.664 (2)	0.118 (3)	0.1438 (7)	0.018*	
C1	1.2275 (6)	0.3148 (4)	0.08300 (8)	0.0138 (8)	
C2	1.4066 (6)	0.4158 (4)	0.09534 (8)	0.0175 (8)	
H2A	1.4062	0.4477	0.1175	0.021*	
C3	1.5882 (6)	0.4694 (4)	0.07423 (8)	0.0193 (9)	
H3B	1.7151	0.5378	0.0822	0.023*	
C4	1.5863 (7)	0.4244 (4)	0.04181 (9)	0.0237 (9)	
H4A	1.7092	0.4635	0.0275	0.028*	
C5	1.4054 (6)	0.3227 (4)	0.03045 (8)	0.0239 (9)	
H5A	1.4044	0.2909	0.0082	0.029*	
C6	1.2250 (6)	0.2665 (4)	0.05106 (7)	0.0186 (8)	
H6A	1.1010	0.1954	0.0432	0.022*	
C7	0.8931 (6)	0.4166 (4)	0.18657 (8)	0.0147 (8)	
C8	1.0704 (7)	0.5046 (4)	0.20239 (8)	0.0232 (9)	
H8A	1.2141	0.5378	0.1913	0.028*	
C9	1.0358 (7)	0.5446 (4)	0.23511 (8)	0.0270 (9)	
H9A	1.1575	0.6045	0.2465	0.032*	
C10	0.8268 (7)	0.4975 (4)	0.25076 (9)	0.0277 (10)	
H10A	0.8030	0.5250	0.2730	0.033*	
C11	0.6510 (7)	0.4101 (4)	0.23415 (8)	0.0220 (9)	
H11A	0.5062	0.3777	0.2451	0.026*	
C12	0.6827 (6)	0.3689 (4)	0.20180 (8)	0.0170 (8)	
H12A	0.5611	0.3090	0.1904	0.020*	
C13	0.6168 (7)	−0.1630 (4)	0.18811 (7)	0.0273 (10)	
H13A	0.4918	−0.2374	0.1966	0.041*	
H13B	0.5715	−0.0493	0.1930	0.041*	
H13C	0.7740	−0.1882	0.1983	0.041*	
C14	0.6370 (6)	−0.1851 (4)	0.15156 (7)	0.0213 (9)	
H14A	0.4814	−0.1515	0.1413	0.026*	
H14B	0.6628	−0.3028	0.1467	0.026*	
C15	0.8451 (6)	−0.0856 (4)	0.13654 (7)	0.0130 (7)	
H15A	1.0005	−0.1226	0.1469	0.016*	
C16	0.8667 (6)	−0.1162 (4)	0.10011 (8)	0.0131 (8)	
C17	1.0577 (6)	−0.2086 (4)	0.08788 (7)	0.0175 (8)	
H17A	1.1767	−0.2512	0.1024	0.021*	
C18	1.0790 (6)	−0.2405 (4)	0.05478 (8)	0.0211 (9)	
H18A	1.2099	−0.3055	0.0468	0.025*	
C19	0.9076 (6)	−0.1766 (4)	0.03360 (8)	0.0213 (9)	
H19A	0.9221	−0.1965	0.0109	0.026*	
C20	0.7162 (7)	−0.0845 (4)	0.04518 (8)	0.0211 (9)	
H20A	0.5981	−0.0413	0.0306	0.025*	
C21	0.6966 (6)	−0.0549 (4)	0.07836 (8)	0.0178 (9)	
H21A	0.5640	0.0086	0.0863	0.021*	

### Atomic displacement parameters (Å^2^)

**Table:**

	*U*^11^	*U*^22^	*U*^33^	*U*^12^	*U*^13^	*U*^23^
P1	0.0134 (4)	0.0160 (4)	0.0139 (5)	−0.0003 (4)	−0.0006 (4)	−0.0004 (4)
O1	0.0146 (12)	0.0300 (15)	0.0122 (11)	−0.0012 (13)	0.0002 (10)	0.0020 (10)
O2	0.0200 (13)	0.0115 (12)	0.0163 (12)	0.0000 (11)	−0.0005 (11)	−0.0006 (10)
O3	0.0136 (12)	0.0178 (13)	0.0191 (13)	−0.0002 (11)	−0.0008 (10)	−0.0001 (11)
N1	0.0132 (14)	0.0114 (15)	0.0119 (16)	0.0030 (13)	−0.0001 (14)	−0.0015 (13)
C1	0.0098 (18)	0.013 (2)	0.018 (2)	0.0016 (16)	0.0051 (14)	0.0038 (16)
C2	0.0175 (19)	0.0173 (18)	0.018 (2)	0.0038 (17)	0.0013 (16)	−0.0023 (16)
C3	0.0152 (19)	0.0126 (19)	0.030 (2)	−0.0028 (17)	−0.0017 (17)	0.0017 (17)
C4	0.020 (2)	0.022 (2)	0.029 (2)	0.0019 (18)	0.0120 (18)	0.0046 (19)
C5	0.028 (2)	0.031 (2)	0.0119 (19)	0.0009 (19)	0.0064 (16)	−0.0006 (17)
C6	0.0222 (19)	0.018 (2)	0.0157 (19)	−0.0053 (18)	−0.0017 (16)	0.0002 (16)
C7	0.0164 (19)	0.0115 (18)	0.0162 (19)	0.0029 (17)	−0.0031 (16)	−0.0003 (16)
C8	0.019 (2)	0.0183 (19)	0.032 (2)	−0.0011 (19)	0.0001 (19)	−0.0022 (17)
C9	0.027 (2)	0.025 (2)	0.029 (2)	0.000 (2)	−0.009 (2)	−0.0110 (18)
C10	0.036 (3)	0.030 (2)	0.017 (2)	0.010 (2)	−0.007 (2)	−0.0079 (19)
C11	0.024 (2)	0.027 (2)	0.015 (2)	0.003 (2)	0.0002 (17)	0.0016 (18)
C12	0.0167 (19)	0.016 (2)	0.019 (2)	−0.0012 (17)	−0.0067 (16)	−0.0012 (16)
C13	0.037 (2)	0.025 (2)	0.020 (2)	−0.0053 (19)	0.0051 (18)	0.0021 (18)
C14	0.028 (2)	0.0157 (19)	0.020 (2)	−0.0020 (17)	−0.0016 (16)	−0.0034 (17)
C15	0.0115 (16)	0.0149 (18)	0.0128 (18)	0.0017 (15)	−0.0032 (16)	0.0010 (17)
C16	0.0141 (18)	0.0083 (18)	0.017 (2)	−0.0031 (16)	−0.0005 (15)	−0.0012 (15)
C17	0.0180 (18)	0.0196 (18)	0.0148 (18)	−0.0021 (19)	−0.0024 (15)	−0.0027 (17)
C18	0.019 (2)	0.019 (2)	0.025 (2)	−0.0018 (18)	0.0042 (16)	−0.0069 (17)
C19	0.033 (2)	0.021 (2)	0.0102 (18)	−0.0100 (18)	0.0022 (16)	−0.0018 (16)
C20	0.029 (2)	0.020 (2)	0.014 (2)	−0.0004 (19)	−0.0041 (18)	0.0012 (17)
C21	0.021 (2)	0.016 (2)	0.016 (2)	0.0027 (17)	−0.0030 (17)	−0.0042 (16)

### Geometric parameters (Å, °)

**Table:**

P1—O3	1.465 (2)	C10—C11	1.380 (5)
P1—O1	1.582 (2)	C10—H10A	0.9500
P1—O2	1.586 (2)	C11—C12	1.384 (4)
P1—N1	1.613 (3)	C11—H11A	0.9500
O1—C1	1.400 (3)	C12—H12A	0.9500
O2—C7	1.417 (4)	C13—C14	1.519 (4)
N1—C15	1.469 (4)	C13—H13A	0.9800
N1—H1	0.838 (10)	C13—H13B	0.9800
C1—C6	1.372 (4)	C13—H13C	0.9800
C1—C2	1.378 (4)	C14—C15	1.530 (4)
C2—C3	1.392 (4)	C14—H14A	0.9900
C2—H2A	0.9500	C14—H14B	0.9900
C3—C4	1.384 (4)	C15—C16	1.525 (4)
C3—H3B	0.9500	C15—H15A	1.0000
C4—C5	1.374 (4)	C16—C17	1.385 (4)
C4—H4A	0.9500	C16—C21	1.386 (4)
C5—C6	1.382 (4)	C17—C18	1.392 (4)
C5—H5A	0.9500	C17—H17A	0.9500
C6—H6A	0.9500	C18—C19	1.384 (4)
C7—C12	1.370 (4)	C18—H18A	0.9500
C7—C8	1.373 (4)	C19—C20	1.376 (5)
C8—C9	1.398 (4)	C19—H19A	0.9500
C8—H8A	0.9500	C20—C21	1.391 (4)
C9—C10	1.370 (5)	C20—H20A	0.9500
C9—H9A	0.9500	C21—H21A	0.9500
			
O3—P1—O1	113.67 (13)	C10—C11—H11A	119.5
O3—P1—O2	116.68 (12)	C12—C11—H11A	119.5
O1—P1—O2	99.51 (12)	C7—C12—C11	118.5 (3)
O3—P1—N1	114.72 (13)	C7—C12—H12A	120.7
O1—P1—N1	105.78 (13)	C11—C12—H12A	120.7
O2—P1—N1	104.81 (13)	C14—C13—H13A	109.5
C1—O1—P1	128.3 (2)	C14—C13—H13B	109.5
C7—O2—P1	121.73 (19)	H13A—C13—H13B	109.5
C15—N1—P1	120.9 (2)	C14—C13—H13C	109.5
C15—N1—H1	113 (2)	H13A—C13—H13C	109.5
P1—N1—H1	121 (2)	H13B—C13—H13C	109.5
C6—C1—C2	122.1 (3)	C13—C14—C15	113.1 (3)
C6—C1—O1	115.6 (3)	C13—C14—H14A	109.0
C2—C1—O1	122.3 (3)	C15—C14—H14A	109.0
C1—C2—C3	117.9 (3)	C13—C14—H14B	109.0
C1—C2—H2A	121.1	C15—C14—H14B	109.0
C3—C2—H2A	121.1	H14A—C14—H14B	107.8
C4—C3—C2	120.9 (3)	N1—C15—C16	113.0 (3)
C4—C3—H3B	119.6	N1—C15—C14	109.0 (3)
C2—C3—H3B	119.6	C16—C15—C14	111.6 (3)
C5—C4—C3	119.6 (3)	N1—C15—H15A	107.7
C5—C4—H4A	120.2	C16—C15—H15A	107.7
C3—C4—H4A	120.2	C14—C15—H15A	107.7
C4—C5—C6	120.5 (3)	C17—C16—C21	118.0 (3)
C4—C5—H5A	119.7	C17—C16—C15	120.3 (3)
C6—C5—H5A	119.7	C21—C16—C15	121.6 (3)
C1—C6—C5	119.1 (3)	C16—C17—C18	121.4 (3)
C1—C6—H6A	120.5	C16—C17—H17A	119.3
C5—C6—H6A	120.5	C18—C17—H17A	119.3
C12—C7—C8	121.9 (3)	C19—C18—C17	119.3 (3)
C12—C7—O2	119.9 (3)	C19—C18—H18A	120.3
C8—C7—O2	118.0 (3)	C17—C18—H18A	120.3
C7—C8—C9	118.8 (4)	C20—C19—C18	120.4 (3)
C7—C8—H8A	120.6	C20—C19—H19A	119.8
C9—C8—H8A	120.6	C18—C19—H19A	119.8
C10—C9—C8	120.1 (4)	C19—C20—C21	119.6 (3)
C10—C9—H9A	120.0	C19—C20—H20A	120.2
C8—C9—H9A	120.0	C21—C20—H20A	120.2
C9—C10—C11	119.7 (3)	C16—C21—C20	121.3 (3)
C9—C10—H10A	120.1	C16—C21—H21A	119.3
C11—C10—H10A	120.1	C20—C21—H21A	119.3
C10—C11—C12	120.9 (4)		

### Hydrogen-bond geometry (Å, °)

**Table:**

*D*—H···*A*	*D*—H	H···*A*	*D*···*A*	*D*—H···*A*
N1—H1···O3^i^	0.84 (1)	2.25 (1)	3.077 (3)	167 (3)

Symmetry codes: (i) *x*−1, *y*, *z*.
